# *Sarm1* deletion suppresses TDP-43-linked motor neuron degeneration and cortical spine loss

**DOI:** 10.1186/s40478-019-0800-9

**Published:** 2019-10-28

**Authors:** Matthew A. White, Ziqiang Lin, Eugene Kim, Christopher M. Henstridge, Emiliano Pena Altamira, Camille K. Hunt, Ella Burchill, Isobel Callaghan, Andrea Loreto, Heledd Brown-Wright, Richard Mead, Camilla Simmons, Diana Cash, Michael P. Coleman, Jemeen Sreedharan

**Affiliations:** 1 Department of Basic and Clinical Neuroscience, The Maurice Wohl Clinical Neuroscience Institute, Institute of Psychiatry, Psychology and Neuroscience (IoPPN), King’s College London, London, SE5 9RT UK; 2 West China School of Medicine, West China Hospital, Sichuan University, Chengdu, China; 3 BRAIN Centre (Biomarker Research And Imaging for Neuroscience), Department of Neuroimaging, IoPPN, King’s College London, London, UK; 4 Division of Systems Medicine, School of Medicine, University of Dundee, Dundee, Scotland UK; 5 John van Geest Centre for Brain Repair, Department of Clinical Neurosciences, University of Cambridge, Cambridge, UK; 6 Department of Neuroscience, Sheffield Institute for Translational Neuroscience, University of Sheffield, Sheffield, UK; 7 Signalling Programme, Babraham Institute, Babraham Research Campus, Cambridge, UK

**Keywords:** Sterile alpha and TIR motif-containing protein 1, Amyotrophic lateral sclerosis, TAR DNA-binding protein 43, Wallerian degeneration, Axonal protection, Dendritic spines

## Abstract

Amyotrophic lateral sclerosis (ALS) is a fatal neurodegenerative condition that primarily affects the motor system and shares many features with frontotemporal dementia (FTD). Evidence suggests that ALS is a ‘dying-back’ disease, with peripheral denervation and axonal degeneration occurring before loss of motor neuron cell bodies. Distal to a nerve injury, a similar pattern of axonal degeneration can be seen, which is mediated by an active axon destruction mechanism called Wallerian degeneration. Sterile alpha and TIR motif-containing 1 (*Sarm1*) is a key gene in the Wallerian pathway and its deletion provides long-term protection against both Wallerian degeneration and Wallerian-like, non-injury induced axonopathy, a retrograde degenerative process that occurs in many neurodegenerative diseases where axonal transport is impaired. Here, we explored whether Sarm1 signalling could be a therapeutic target for ALS by deleting *Sarm1* from a mouse model of ALS-FTD, a TDP-43^Q331K^, YFP-H double transgenic mouse. *Sarm1* deletion attenuated motor axon degeneration and neuromuscular junction denervation. Motor neuron cell bodies were also significantly protected. Deletion of *Sarm1* also attenuated loss of layer V pyramidal neuronal dendritic spines in the primary motor cortex. Structural MRI identified the entorhinal cortex as the most significantly atrophic region, and histological studies confirmed a greater loss of neurons in the entorhinal cortex than in the motor cortex, suggesting a prominent FTD-like pattern of neurodegeneration in this transgenic mouse model. Despite the reduction in neuronal degeneration, *Sarm1* deletion did not attenuate age-related behavioural deficits caused by TDP-43^Q331K^. However, *Sarm1* deletion was associated with a significant increase in the viability of male TDP-43^Q331K^ mice, suggesting a detrimental role of Wallerian-like pathways in the earliest stages of TDP-43^Q331K^-mediated neurodegeneration. Collectively, these results indicate that anti-SARM1 strategies have therapeutic potential in ALS-FTD.

**Electronic supplementary material:**

The online version of this article (10.1186/s40478-019-0800-9) contains supplementary material, which is available to authorized users.

## Introduction

Amyotrophic lateral sclerosis (ALS) is a progressive and ultimately fatal adult motor neuron disease that causes inexorable paralysis of limb, bulbar and respiratory muscles. Patients may also demonstrate cognitive deficits in keeping with frontotemporal dementia (FTD). Current disease modifying approaches for ALS have only a modest effect on survival and new therapeutic agents are urgently required. Targeting the earliest stages in the neurodegenerative process holds the greatest promise for therapeutic advances.

In ALS, pathological studies demonstrate early peripheral denervation before ventral nerve root or motor neuronal cell body loss suggesting that degeneration starts at the nerve terminal and progresses retrogradely along the axon [20]. Indeed, distal corticospinal tract inflammatory changes [8, 37] and giant axonal swellings have been detected in spinal cords from ALS patients, suggesting early distal axonal degeneration [15, 53]. Similarly, mutant-SOD1 mice with ALS-like phenotypes exhibit presymptomatic muscle denervation and terminal axonal degeneration before anterior horn cell loss [20, 58]. Axonal transport also appears to fail early in ALS. This is suggested by accumulation of ubiquitinated proteins, phosphorylated neurofilaments, mitochondria and microtubules in proximal axons and anterior horn cells of ALS patients [83]. Mutations in proteins with axonal functions are linked to ALS, including SMN, dynactin and spatacsin [14, 54]. Given that the axon constitutes 99.9% of the volume of a motoneuron and therefore places great metabolic demands on the cell, it is perhaps unsurprising that axonal degeneration can be an early event in ALS, and thus an attractive target for therapeutic intervention.

Almost all ALS and as many as half of FTD cases are characterised by pathological ubiquitinated inclusions of TAR DNA-binding protein 43 kDa (TDP-43) [3, 52]. The identification of mutations of TDP-43 in patients with ALS and FTD demonstrates that TDP-43 plays mechanistic roles in neurodegeneration [2, 7, 39]. Disturbances in TDP-43 homeostasis have been shown to affect axonal function and TDP-43 aggregates may form early within motor axons [10]. Knocking down or overexpressing wild-type or mutant TDP-43 disrupts motor neuron axons and terminal arborisations in flies [16, 42, 44] and zebrafish [38, 41]. TDP-43 transgenic rodents demonstrate early changes in neuromuscular junction (NMJ) and axonal integrity [65, 73, 81, 85, 87]. TDP-43 also localises within presynaptic vesicles in motoneurons in human spinal cord [62] and in axons in vitro [38]. Furthermore, axonal injury causes striking redistribution of TDP-43 from the nucleus to the cytoplasm and axon [50, 63]. Collectively, these results highlight how aberrant homeostasis of TDP-43 can directly impair axonal physiology, potentially causing neurodegeneration.

Given the importance of axon degeneration in ALS, there has been great interest in trying to protect axons and synapses as a therapeutic approach. Following injury to a nerve, typically a cut or crush, the process of Wallerian degeneration ensues, leading to fragmentation of axon fibres distal to the injury site within 72 h. This fragmentation was long thought to be due to loss of trophic support from the cell body [80], but studies of the mutant mouse *Wld*^*S*^ (Wallerian degeneration slow) established Wallerian degeneration as a tightly regulated process separate and distinct from apoptosis of the cell body [46]. While wild-type axons start to degenerate from 36 h following axotomy, *Wld*^*S*^ axons remain intact for weeks and can still conduct action potentials [46]. *Wld*^*S*^ encodes a fusion protein with nicotinamide mononucleotide adenylyltransferase 1 (NMNAT1) activity, which compensates for the loss of the axonal NMNAT2 isoform, which has a short half-life and is rapidly depleted from axonal segments distal to the site of injury or when its supply is interrupted for other reasons such as axonal transport deficit [13, 25, 47].

Importantly, screening in *Drosophila* has identified Wallerian degeneration regulating genes, indicating the presence of an endogenous axonal auto-destruction pathway that is conserved in mammals [51, 55, 84]. The first of these genes to be identified, sterile alpha and TIR motif-containing 1 (encoding Sarm1), acts downstream of NMNAT2 loss to promote axon degeneration following axotomy [24, 26, 45, 55, 79]. In fact, the deletion of *Sarm1* is significantly more protective than *Wld*^*S*^ overexpression in an *Nmnat2* depletion model of neurodegeneration as mice age [27]. These observations confirmed that Wallerian degeneration is an active, genetically programmed process that can be potently inhibited.

Evidence to suggest that Wallerian-like processes occur in neurodegenerative diseases comes from recent studies in which the axon outgrowth and regeneration factor Stathmin 2 (also known as SCG10) was found to be downregulated in ALS spinal motor neurons [40, 49]. Loss of Stathmin 2 was previously shown to enhance Wallerian degeneration following axon transection [66]. Furthermore, impaired axonal mitochondrial function, an early pathophysiological event in ALS [67], activates the Wallerian pathway leading to Sarm1-dependent axonal degeneration [72]. Mechanistic studies have also shown, to varying degrees, that axonal protection can be neuroprotective. For example, mice lacking *Sarm1* have improved functional outcomes as well as attenuated axonal injury following mild traumatic brain injury [31], while deletion of *Sarm1* prevents chemotherapy induced peripheral neuropathy [23]. *Wld*^*S*^ can ameliorate axonopathy in models of Charcot-Marie-Tooth disease, Parkinson’s disease and glaucoma [5, 60, 61]. *Wld*^*S*^ is also protective in the progressive motor neuronopathy *(pmn)* mouse [18]. Although *Wld*^*S*^ has little effect on survival in mutant-SOD1 mice, it significantly protects NMJs in young G93A transgenic mice [19, 77]. Studies in *C. elegans* demonstrate that loss of the *Sarm1* homolog *Tir-1* suppresses neurodegeneration and delays paralysis induced by mutant TDP-43 [78]. Finally, the human *SARM1* locus has also been associated with sporadic ALS risk [22]. Collectively, these observations suggest that Wallerian-like mechanisms could contribute to the neurodegeneration seen in motor neuron diseases, and that depletion of SARM1 could have therapeutic potential in ALS. However, there have been no studies in mammalian models that have investigated a link between Wallerian pathways and TDP-43-mediated neurodegeneration. This is a particularly important question as TDP-43 pathology is a hallmark of 98% of ALS, including sporadic ALS. We therefore sought to determine whether SARM1 signalling could be a therapeutic target in ALS by deleting *Sarm1* from a TDP-43^Q331K^ transgenic mouse model of ALS-FTD. Our results demonstrate that *Sarm1* deletion has a neuroprotective effect and leads to both improvements in motor axonal integrity and, importantly, lumbar motor neuron survival.

## Materials and methods

### Mouse breeding and maintenance

**Fig. 1:**
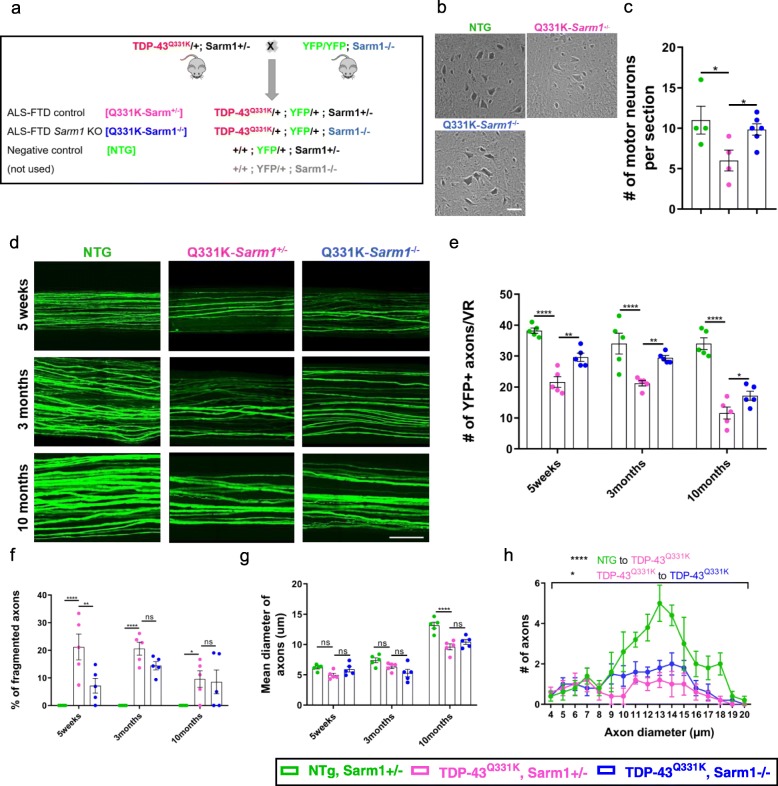
*Sarm1* deletion mitigates TDP43^Q331K^-mediated motor neuron loss and axon degeneration. **a**. Breeding scheme. **b**. Nissl-stained lumbar motor neurons of 10-month-old mice. Representative images shown. Scale bar, 50 μm. **c**. Quantification of Nissl-stained lumbar motor neurons per section at spinal cord L3. (*n* = 4 NTG; *n* = 4 Q331K-*Sarm1*^+/−^; *n* = 6 Q331K-*Sarm1*^−/−^) ANOVA *P* = 0.0404. Pairwise comparisons: NTG vs. Q331K-*Sarm1*^+/−^: * *P* = 0.0351; Q331K-*Sarm1*^+/−^ vs. Q331K-*Sarm1*^−/−^: * *P* = 0.0392. **d**. Representative images showing intact YFP+ axons of L4 ventral roots from mice of three genotypes at different time points. Scale bar 100 μm. **e**. Quantification of fluorescent axons in wholemount YFP+ L4 ventral roots at different time points. ANOVA Interaction *P* = 0.0157. Pairwise comparisons: 5 weeks, Q331K-*Sarm1*^+/−^ vs. Q331K-*Sarm1*^−/−^: ** *P* = 0.0026; 3 months, Q331K-*Sarm1*^+/−^ vs. Q331K-*Sarm1*^−/−^: ** *P* = 0.0040; 10 months, Q331K-*Sarm1*^+/−^ vs. Q331K-*Sarm1*^−/−^: * *P* = 0.0289. **f**. Percentage of YFP+ axons undergoing fragmentation in L4 ventral roots at different time points. ANOVA Genotype *P* < 0.0001. Pairwise comparisons: 5 weeks, Q331K-*Sarm1*^+/−^ vs. Q331K-*Sarm1*^−/−^: ** *P* = 0.0014; 3 months, Q331K-*Sarm1*^+/−^ vs. Q331K-*Sarm1*^−/−^: ns *P* = 0.1104; 10 months, NTG vs. Q331K-*Sarm1*^+/−^: * *P* = 0.0464; Q331K-*Sarm1*^+/−^ vs. Q331K-*Sarm1*^−/−^: ns *P* = 0.7870. **g**. Fibre diameters measured at the thickest part of intact YFP+ axons in L4 ventral roots at different time points. ANOVA Interaction *P* = 0.0051. Pairwise comparisons: 5 weeks, NTG vs. Q331K-*Sarm1*^+/−^: ns *P* = 0.1295; Q331K-*Sarm1*^+/−^ vs. Q331K-*Sarm1*^−/−^: ns *P* = 0.2198; 3 months, NTG vs. Q331K-*Sarm1*^+/−^: ns *P* = 0.1567; Q331K-*Sarm1*^+/−^ vs. Q331K-*Sarm1*^−/−^: ns *P* = 0.1567; 10 months, Q331K-*Sarm1*^+/−^ vs. Q331K-*Sarm1*^−/−^: ns *P* = 0.1810. **h**. Distribution of diameters of YFP+ L4 motor axons at 10 months of age. ANOVA Interaction *P* = 0.0014. Pairwise comparisons: Q331K-*Sarm1*^+/−^ vs. Q331K-*Sarm1*^−/−^: * *P* = 0.0386. For (**e**-**h**) (*n* = 5 mice per genotype); ****P < 0.0001. For (**c**) one-way (**e**-**h**) two-way ANOVA followed by Holm-Sidak *post-hoc* test for pairwise comparisons. Error bars represent mean ± s.e.m

High expression hTDP^Q331K^ and *Sarm1* knock out mice were acquired from Jackson Laboratories and maintained on a C57BL/6Babr background in a 12 h light/dark cycle with ad libitum access to food and water. Mice were housed in Tecniplast cages within a clean facility. Individual cages contained environmental enrichment items and group sizes of 2–5 mice were routinely maintained. Transgenic colonies were generated by breeding male mice heterozygous for both *Sarm1* deletion and TDP^Q331K^ transgenes with *Sarm1* heterozygous or null females. Crosses were designed such that all mice also expressed the Thy1-YFP transgene (see Fig. 1a) (Feng Neuron 2000). Genotyping for the YFP-H, TDP^Q331K^ and *Sarm1* knockout alleles was performed as described previously [1, 4, 26]. Both male and female mice were used for experimental studies; mouse sex and age are highlighted where appropriate. All animal experiments were performed under the UK animals (Scientific Procedures) Act 1986 Amendment Regulations 2012 on Project Licence 70/7620.

### Behavioural testing

Motor testing was performed using Rotarod on both male and female mice (Ugo Basile, Model 7650, Varese, Italy). At least 24 h prior to testing mice were first trained for 5 min at the slowest speed and then 7 min with acceleration. During testing mice were subjected to 7 min trials with acceleration from 3.5 to 35 rpm. In each session mice were tested 3 times with a trial separation of 30 min. The latency to fall (maximum 420 s) for each mouse was recorded and mean values for each mouse calculated. An individual mouse recording was excluded if it fell off the rod while moving backwards, accidentally slipped or jumped off at a slow speed. Two consecutive passive rotations were counted as a fall and the time recorded as the end point for that mouse. Mouse weights were recorded immediately after completion of rotarod testing. All testing was conducted by operators who were blind to genotype and in a randomised order.

To assess clasping, male mice were suspended by the base of the tail and observed for 10–15 s. Testing took place immediately following measurement of weight. Hindlimb clasping was rated from 0 to 4 based on severity: 0 = hindlimbs splayed outward and away from the abdomen, 1 = one hindlimb partially retracted inwards towards the abdomen for at least 50% of the observation period, 2 = both hindlimbs partially retracted inwards towards the abdomen for at least 50% of the observation period, 3 = one hindlimb fully retracted inwards towards the abdomen for at least 50% of the observation period 4 = both hindlimbs fully retracted inwards towards the abdomen for at least 50% of the observation period.

All marble-burying testing was conducted in the morning and blind to genotype on both male and female mice. Cages of size 39.1 cm × 19.9 cm × 16.0 cm height (Tecniplast) were used. Fresh bedding material (Datesand, grade 6) was placed into each cage to a height of ~ 6 cm. Ten glass marbles (1 cm) were placed evenly across the bedding. Ten cages were prepared in a single round. One mouse was placed in each of the cages and the lids replaced. Mice were left undisturbed for 30 min under white light. Mice were then removed, and the number of marbles buried by at least two thirds was scored. Cages were reset using the same bedding material to test another 10 mice.

### Pathological studies

Unless specified, mice were culled by asphyxiation with CO_2_ followed by cervical dislocation and tissue extraction. Brains and gastrocnemius muscles of male and female mice were weighed. The tissues were then immersion fixed in 4% paraformaldehyde (PFA) at 4 °C for 24–48 h, washed in PBS, cryoprotected in 30% sucrose in PBS to store at 4 °C. For ex vivo MRI studies animals were anaesthetised and transcardially perfused with PBS followed by a 4% PFA solution. Heads were removed, scalped and then placed into 4% PFA for at least 24 h. Heads were subsequently rehydrated in PBS with 0.05% sodium azide for at least 14 days prior to MRI scanning. For lumbar motor neuron quantification tissues were used following transcardial perfusion as above.

### YFP-H motor axon quantification

Spinal cords were extracted from vertebral columns and L4 ventral nerve roots were carefully dissected from male mice. Following dissection the nerve roots were treated for wholemount fluorescence preparation using Vectashield Mounting Medium (Vector Laboratories) as described previously [6]. Z-stacks of the wholemount YFP-H axons were obtained using a Nikon A1R Confocal Laser Microscope System with a 20x objective. The number of YFP+ axons per roots, axon diameters and percentage of fragmentated axons were then measured using NIS-Elements imaging software blind to genotype.

### Quantification of neuromuscular junction innervation

Fixed, cryoprotected gastrocnemius muscles from male mice were placed in a silicone mould with M1 matrix (Thermofisher Scientific), and frozen on dry ice. Longitudinal cryosections (50 μm) were mounted onto slides, air dried at room temperature (R/T) for 5 min and stored at − 80 °C. To stain neuromuscular junctions (NMJs), slides were brought up to R/T and incubated in blocking solution (2% BSA, 0.5% Triton X-100, 0.1% sodium azide) for 1 h. Primary antibodies against βIII-tubulin (rabbit polyclonal, Sigma T2200) and synaptophysin (mouse monoclonal, Abcam ab8049) were applied at a 1:200 dilution in blocking solution. Sections were incubated at R/T overnight. Sections were washed in PBS and incubated for 90 min with mouse and rabbit Alexa488-conjugated secondary antibodies (Thermofisher Scientific) diluted 1:500 in blocking solution together with TRITC-conjugated alpha bungarotoxin (Sigma, T0195). Z-stacks were obtained using an Olympus Whole Slide Scanner (VS120) with a 20x objective. NMJs from flattened z-stacks of muscle were analysed blind to genotype. Brightness and contrast thresholds were set to optimise the signal-to-noise ratio of the presynaptic staining (anti-tubulin and anti-synaptophysin). To assess the co-localization of pre- and post-synaptic membrane proteins at the NMJ, 90–110 NMJs were analysed per animal per genotype for all ages by eye. Fully innervated NMJs were defined as demonstrating full co-localization of pre- and post- synaptic staining. Fully denervated NMJs were defined as alpha-bungarotoxin signal in the absence of pre-synaptic staining. Partially innervated NMJs were defined as having partial overlap of pre- and post-synaptic labelling.

### Spinal cord motor neuron Nissl staining

Fixed spinal cords from male mice were sub-dissected, paraffin embedded and subsequently sectioned (10 μm thickness) onto charged slides. Sections were initially dewaxed in xylene followed by rehydration through graded alcohols and then washed in water. Sections were stained with cresyl etch violet (0.05%) for 30 min, briefly washed in 96% ethanol, dehydrated in 100% ethanol, cleared in xylene, mounted (Permount, Fisher) and dried overnight at R/T. Images were taken on an EVOS FL Cell Imaging System (Thermofisher Scientific) using a 20x objective. For motor neuron quantification, Nissl positive cells of the ventral horn ≥20 μm in diameter were counted from 7 to 8 sections per mouse to determine the average count per section.

### Cortical neuron quantification

Cyroprotected mouse brains from male mice were embedded and frozen in M1 matrix and sectioned coronally at 60 μm thickness on a cryostat (Leica Biosystems). Sections were then mounted on slides and coverslips applied. Z-stacks were acquired using an Olympus Whole Slide Scanner (VS120) with a 20x objective. Coronal sections from flattened z-stacks of brains were analysed using ImageJ blind to genotype. YFP+ cortical neurons from primary motor cortex and entorhinal cortex were measured from matching sections of each mouse from each genotype. Consistent regions of interest were drawn around the cortex and cells counted using Visiopharm image analysis software (Hoersholm, Denmark).

### Cortical dendritic spine analysis

YFP-H fluorescence was captured using the Nikon iSIM Super Resolution Microscope System running Nikon Elements software using a GFP filter. We focused on the distal branches of the apical dendrites 120 μm from the cell bodies of the neurons in layer V of the motor cortex. To image apical dendritic spines, a 100x oil objective was used to acquire z-plane images with 0.8 μm intervals through 20 μm of tissue. Five images were collected from the primary motor cortex per animal. To characterise the apical dendritic spines, z-stack images were analysed using Neurolucida Explorer software (MBF Bioscience, USA). Dendrites were traced through stacks with spines marked, and images then exported to Neurolucida Explorer for spine quantification. Branched structure analysis was used to analyse the number of dendritic spines per μm and density of spines of different morphologies. Spines were classified either as mushroom, stubby or thin type according to spine neck length and spine head size, referring to established parameters [30].

### Structural magnetic resonance imaging

Ex vivo, in loco MRI was performed for 10-month-old female mice (*n* = 32) using a 9.4 T horizontal bore BioSpec 94/20 scanner (Bruker). The mouse heads were placed four at a time in a 50 ml Falcon tube filled with fomblin (Solvay) and scanned overnight using a quadrature birdcage transceiver coil (39 mm internal diameter). T2-weighted images were acquired using a 3D fast spin-echo sequence: effective echo time 30 ms, repetition time 3000 ms, field of view 25 × 25 × 20 mm, acquisition matrix 250 × 250 × 200. Diffusion tensor imaging (DTI) data was acquired using a 2D Stejskal-Tanner spin-echo sequence: echo time 22.6 ms, repetition time 4000 ms, field of view 25.6 × 25.6 mm, acquisition matrix 256 × 256, 67 slices with 0.2 mm thickness and 0.1 mm gap, b-value 1500, 30 diffusion directions, 4 b0 images. Fractional anisotropy (FA) maps were computed from the DTI data using dtifit (FSL).

A study-specific template was generated from a subset of 20 mice (antsMultivariateTemplateConstruction2.sh), and all brains were then registered to the template (antsRegistrationSyN.sh). Jacobian determinant maps of the deformation matrices were computed and log-transformed to perform voxel-wise nonparametric statistics (FSL randomise, 5000 permutations, threshold-free cluster enhancement) to compare local brain volumes between NTG, Q331K-Sarm1^+/−^, and Q331K-Sarm1^−/−^ mice.

To perform region-of-interest (ROI) analysis, the study-specific template was registered to the DSURQE mouse brain atlas (Mouse Imaging Centre, Toronto), which consists of 28 bilateral and 154 unilateral ROIs (336 total). These labels were transformed to the study-specific template space, and the volume of each ROI in each mouse was calculated. DSURQE mouse brain atlas can be accessed at: https://wiki.mouseimaging.ca/display/MICePub/Mouse+Brain+Atlases#MouseBrainAtlases-Dorr-Steadman-Ullmann-Richards-Qiu-Egan(40 μm,DSURQE).

### Randomisation

The order and genotype of animals and samples tested was randomized by one operator before subsequent experimental studies were conducted by a second investigator.

### Experimental design and statistical analysis

Experimental data were conducted by researchers blinded to the genotype of animals. Statistical analyses were conducted using Prism 8.1.2 (GraphPad). Graphs were plotted using GraphPad. Use of parametric tests required data to be sampled from a Gaussian distribution. Homogeneity of variance between experimental groups was confirmed by the Browne-Forsythe test for ANOVA. For comparisons between genotypes or experimental groups, one-way or two-way ANOVA was used. Multiple comparisons by ANOVA were corrected using the Holm–Sidak test. All statistical comparisons are based on biological replicates unless stated otherwise. Where technical replication of experiments occurs, this is highlighted in the respective method. Behavioural testing by rotarod and hind limb clasping as well as weight were analysed by Mixed-effects analysis with Geisser-Greenhouse correction followed by Holm-Sidak multiple comparison test. Unless stated otherwise, all charts show mean ± s.e.m. and statistical tests used are described in the relevant results or figure legends. *P*-values < 0.05 were considered significant for all statistical analyses used.

### Data availability

The authors will make all data available to readers upon reasonable request.

## Results

### *Sarm1* deletion significantly reduces TDP-43^Q331K^-mediated motor neuron degeneration

To determine whether *Sarm1* deletion could attenuate TDP-43-mediated neurotoxicity we designed a crossing scheme to knock out *Sarm1* from a transgenic mouse model of ALS that overexpresses human mutant TDP-43^Q331K^ under the mouse prion promoter [4]. The cross was designed such that all study mice and littermate controls were also transgenic for YFP-H to facilitate visualisation of central and peripheral neuronal structures [6, 17]. As *Sarm1*^*+/−*^ mice show no significant protection from Wallerian degeneration at 5d or more post-lesion [55] we used TDP-43^Q331K^-*Sarm1*^*+/−*^ as controls for TDP-43^Q331K^-*Sarm1*^*−/−*^, so that littermate controls could be used without requiring excessively large breeding numbers (Fig. 1a). The study proceeded using three genotypic groups of mice, analysing males and females separately: TDP-43^Q331K^, *Sarm1*^+/−^, YFP-H (hereon referred to as Q331K-*Sarm1*^*+/−*^), TDP-43^Q331K^, *Sarm1*^−/−^, YFP-H (Q331K-*Sarm1*^−/−^), and *Sarm1*^+/−^, YFP-H mice with no TDP-43^Q331K^ transgene (NTG).

To examine if *Sarm1* deletion significantly reduces TDP-43^Q331K^-mediated motor neuron degeneration, Nissl stained cells of the ventral horn greater than 20 μm in diameter were counted (Fig. 1b, c). TDP-43^Q331K^ overexpression caused significant motor neuron loss at 10 months of age, reducing numbers by 46% in Q331K-*Sarm1*^*+/−*^ mice compared to NTG mice (*p* = 0.0351). However, this loss was significantly attenuated in Q331K-*Sarm1*^*−/−*^ mice (*p* = 0.0392), which demonstrated 64% more motor neurons than Q331K-*Sarm1*^*+/−*^ mice (only 11% below the numbers observed in NTG mice). This indicates that *Sarm1* deletion significantly reduces motor neuron degeneration caused by TDP-43^Q331K^.

### *Sarm1* deletion mitigates TDP43^Q331K^-mediated motor axon degeneration

To examine if enhanced motor neuron survival was also associated with preservation of motor axons, a longitudinal histological evaluation was performed by exploiting the sparse labelling of neurons by YFP-H in L4 ventral nerve roots. Intact and fragmented YFP positive (YFP+) axons within the L4 ventral nerve root were quantified at 5 weeks, 3 months and 10 months of age (Fig. 1d-f). While NTG mice demonstrated ~ 30–40 YFP+ motor axons at all time points, with no axons demonstrating fragmentation, Q331K-*Sarm1*^*+/−*^ mice demonstrated only ~ 20 YFP+ axons, ~ 21% of which were fragmented as early as 5 weeks of age (Fig. 1e, f). Furthermore, the number of intact axons in Q331K-*Sarm1*^*+/−*^ mice reduced significantly over time, indicating progressive, age-related axon degeneration due to TDP-43^Q331K^ expression. However, in Q331K-*Sarm1*^*−/−*^ mice axon loss was significantly attenuated at all ages tested, and early fragmentation was reduced by 67% at 5 weeks when compared to Q331K-*Sarm1*^*+/−*^ mice (*p* = 0.0014) (Fig. 1e, f). We also assessed the diameter of remaining unfragmented YFP+ axons within the L4 ventral nerve root. At 10 months of age, the mean axon diameter was significantly reduced in the Q331K-*Sarm1*^*+/−*^ mice by 27% compared to NTG (*p* < 0.0001) (Fig. 1g), and this was partially attenuated in Q331K-*Sarm1*^*−/−*^ mice (*p* = 0.0386) (Fig. 1h). Collectively, these results indicate that *Sarm1* deletion partially protects motor axons from the neurodegenerative effects of human mutant TDP-43^Q331K^.

### *Sarm1* deletion reduces TDP-43^Q331K^-mediated NMJ degeneration

**Fig. 2:**
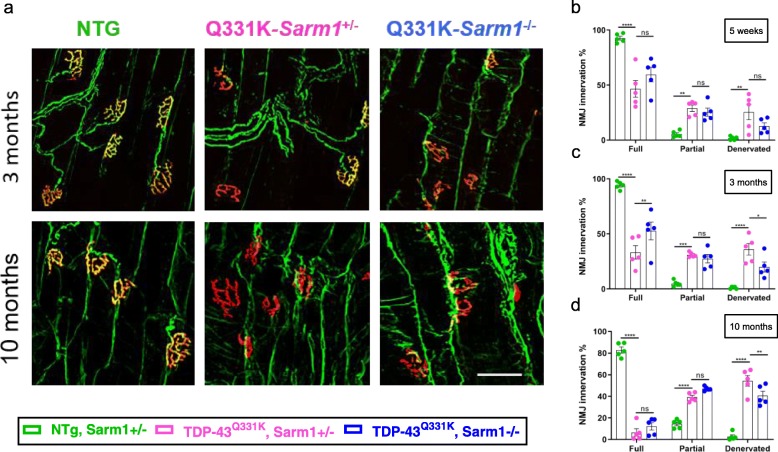
*Sarm1* deletion reduces TDP-43^Q331K^-mediated NMJ degeneration. **a**. Representative images of immunofluorescent staining of NMJs in the gastrocnemius muscle from mice at different time points (Green = synaptophysin and β-III-tubulin co-stain, Red = α-bungarotoxin). Scale bar, 50 μm. (**b**-**d**) Percentages of fully innervated, partially innervated, and denervated NMJs in the gastrocnemius muscles of each genotype at different time points. **b**. At 5 weeks, ANOVA Interaction *P* < 0.0001. Pairwise comparisons: Fully innervated, Q331K-*Sarm1*^+/−^ vs. Q331K-*Sarm1*^−/−^: ns *P* = 0.0520; Partially innervated, NTG vs. Q331K-*Sarm1*^+/−^: ** *P* = 0.0027; Q331K-*Sarm1*^+/−^ vs. Q331K-*Sarm1*^−/−^: ns *P* = 0.5852; Denervated, NTG vs. Q331K-*Sarm1*^+/−^: ** *P* = 0.0024; Q331K-*Sarm1*^+/−^ vs. Q331K-*Sarm1*^−/−^: ns *P* = 0.1107. **c**. At 3 months: ANOVA Interaction *P* < 0.0001. Pairwise comparisons: Fully innervated, Q331K-*Sarm1*^+/−^ vs. Q331K-*Sarm1*^−/−^: ** *P* = 0.0032; Partially innervated, NTG vs. Q331K-*Sarm1*^+/−^: *** *P* = 0.0004; Q331K-*Sarm1*^+/−^ vs. Q331K-*Sarm1*^−/−^: ns *P* = 0.5585; Denervated, Q331K-*Sarm1*^+/−^ vs. Q331K-*Sarm1*^−/−^: * *P* = 0.0124. **d**. At 10 months: ANOVA Interaction *P* < 0.0001. Pairwise comparisons: Fully innervated, Q331K-*Sarm1*^+/−^ vs. Q331K-*Sarm1*^−/−^: ns *P* = 0.1949; Partially innervated, Q331K-*Sarm1*^+/−^ vs. Q331K-*Sarm1*^−/−^: ns *P* = 0.0711; Denervated, Q331K-*Sarm1*^+/−^ vs. Q331K-*Sarm1*^−/−^: ** *P* = 0.0030. For (**b**-**d**) (*n* = 5 mice per genotype); ****P < 0.0001; two-way ANOVA followed by Holm-Sidak *post-hoc* test for pairwise comparisons. Error bars represent mean ± s.e.m

Previous studies have shown that deletion of *Sarm1* preserves NMJ integrity for several days following peripheral nerve transection [55]. We therefore assessed whether *Sarm1* deletion afforded similar protection against TDP-43^Q331K^-mediated NMJ degeneration (Fig. 2a-d). At 5 weeks of age NMJ innervation in gastrocnemius muscles of TDP-43^Q331K^ mice was considerably lower than in NTG mice, indicating that TDP-43^Q331K^ causes very early and severe denervation (*p* < 0.0001) (Fig. 2b). Innervation patterns were comparable between Q331K-*Sarm1*^*+/−*^ and Q331K-*Sarm1*^*−/−*^ mice at 5 weeks of age, but at 3 months of age Q331K-*Sarm1*^*−/−*^ mice demonstrated 45% fewer denervated NMJs (*p* = 0.0124) and 58% more fully-innervated NMJs (*p* = 0.0032) than Q331K-*Sarm1*^*+/−*^ mice (Fig. 2b, c). By 10 months of age the percentage of denervated NMJs was still significantly reduced in Q331K-*Sarm1*^*−/−*^ mice compared to Q331K-*Sarm1*^*+/−*^ mice (*p* = 0.0030), and there was a trend towards an increase in partially innervated NMJs (*p* = 0.0711) (Fig. 2d). To determine if these improvements in NMJ innervation influenced muscle bulk, gastrocnemius muscle weights were compared. Male TDP-43^Q331K^ mice as young as 5 weeks of age showed significant muscle weight loss, which progressed with age (Additional file 1a). In females, significant muscle loss was only observed in TDP-43^Q331K^ mice at 15 months of age (Additional file 1b). However, no significant differences in muscle weight were seen between Q331K-*Sarm1*^*+/−*^ and Q331K-*Sarm1*^*−/−*^ mice, indicating that the improvement in NMJ innervation due to *Sarm1* deletion was not enough to influence muscle atrophy caused by TDP-43^Q331K^.

### TDP-43^Q331K^-mediated cerebral atrophy and cortical neuronal loss are not suppressed by Sarm1 deletion

**Fig. 3:**
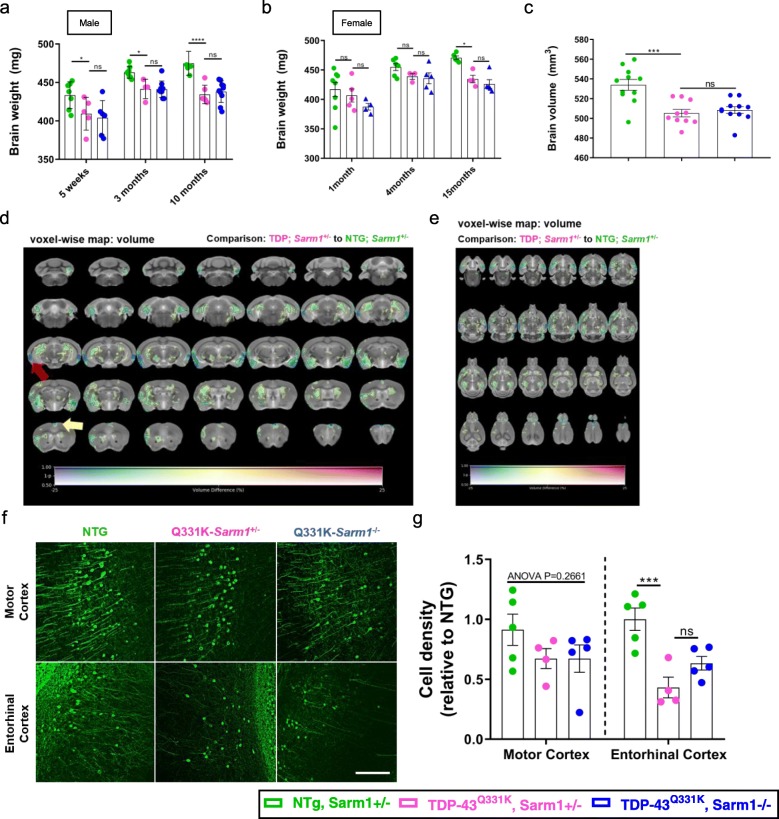
Neurodegeneration is more prominent in the entorhinal cortex than motor cortex of TDP-43^Q331K^ mice. **a**-**b**. Brain weights of mice at different time points. **a**. Male (*n* = 6–8 NTG; *n* = 4–6 Q331K- *Sarm1*^+/−^; *n* = 6–10 Q331K-*Sarm1*^−/−^). ANOVA genotype *P* < 0.0001. Pairwise comparisons: 5 weeks, NTG vs. Q331K-*Sarm1*^+/−^: * *P* = 0.0160; Q331K-*Sarm1*^+/−^ vs. Q331K-*Sarm1*^−/−^: ns *P* = 0.5696; 3 months, NTG vs. Q331K-*Sarm1*^+/−^: * *P* = 0.0458; Q331K-*Sarm1*^+/−^ vs. Q331K-*Sarm1*^−/−^: ns *P* = 0.9658; 10 months, NTG vs. Q331K-*Sarm1*^+/−^: **** *P* < 0.0001; Q331K-*Sarm1*^+/−^ vs. Q331K-*Sarm1*^−/−^: ns *P* = 0.6290. **b**. Female (*n* = 5–8 NTG; *n* = 3–5 Q331K-*Sarm1*^+/−^; *n* = 4–5 Q331K-*Sarm1*^−/−^). ANOVA genotype *P* = 0.0006. Pairwise comparisons: 1 month, NTG vs. Q331K-*Sarm1*^+/−^: ns *P* = 0.3873; Q331K-*Sarm1*^+/−^ vs. Q331K-*Sarm1*^−/−^: ns *P* = 0.3283; 4 months, NTG vs. Q331K-*Sarm1*^+/−^: ns *P* = 0.4957; Q331K-*Sarm1*^+/−^ vs. Q331K-*Sarm1*^−/−^: ns *P* = 0.8347; 15 months, NTG vs. Q331K-*Sarm1*^+/−^: * *P* = 0.0279; Q331K-*Sarm1*^+/−^ vs. Q331K-*Sarm1*^−/−^: ns *P* = 0.5565. **c**. Brain volumes of female mice at 10 months of age measured by ex vivo MRI (*n* = 11 NTG; *n* = 10 Q331K-*Sarm1*^+/−^; *n* = 11 Q331K-*Sarm1*^−/−^). ANOVA *P* < 0.0001. Pairwise comparisons: NTG vs. Q331K-*Sarm1*^+/−^: *** *P* = 0.0001; Q331K-*Sarm1*^+/−^ vs. Q331K-*Sarm1*^−/−^: ns *P* = 0.6180. **d**-**e**. MRI study-specific template (**d**. coronal; **e**. transverse) with an overlay representing voxel-wise volume differences (%) between Q331K-*Sarm1*^*+/−*^ and NTG mice at 10 months of age. The colour of the overlay indicates the inter-group volume difference (warm and cool colours represent gain and loss of volume, respectively, ranging from − 25 to 25%), while the transparency indicates the statistical significance, ranging from FWE-corrected *p* value 0.5 (transparent) to 0 (opaque). Areas in which FWE-corrected *p* value < 0.05 are contoured in black. (Red arrow – entorhinal cortex; yellow arrow – cingulate cortex) **f**-**g**. Representative images of YFP+ neurons from the primary motor cortex (upper) and entorhinal cortex (lower) from mice 10 months of age. Scale bar 200 μm. **f**. Density of YFP+ neurons in both the primary motor and entorhinal cortex (*n* = 5 NTG; *n* = 4 Q331K-*Sarm1*^+/−^; *n* = 5 Q331K-*Sarm1*^−/−^). YFP+ neuron density motor cortex: ANOVA *P* = 0.2661. YFP+ neuron density entorhinal cortex: ANOVA *P* = 0.0013, Pairwise comparisons: NTG vs. Q331K-*Sarm1*^+/−^: *** *P* = 0.0009; Q331K-*Sarm1*^+/−^ vs. Q331K-*Sarm1*^−/−^: ns *P* = 0.1079. (**a**-**b**) Two-way (**c**, **g**) one-way ANOVA followed by Holm-Sidak *post-hoc* test for pairwise comparisons; error bars represent mean ± s.e.m

ALS overlaps clinically, pathologically and genetically with FTD, which is characterised by atrophy of the temporal as well as the frontal lobes of the brain. We therefore examined the brains of our mice to determine if TDP-43^Q331K^ overexpression and/or *Sarm1* play a role in neurodegeneration of cerebral structures. An examination of whole brains from both male and female mice was performed. This demonstrated a significant loss of brain mass in male Q331K-*Sarm1*^*+/−*^ and Q331K-*Sarm1*^*−/−*^ mice compared to NTG from early timepoints (6% at 5 weeks, 5% at 3 months, and 8% at 10 months of age, Fig. 3a). Significant brain weight loss in females was only seen at 15 months of age (Fig. 3b). No significant differences were seen between Q331K-*Sarm1*^*+/−*^ and Q331K-*Sarm1*^*−/−*^ mice, suggesting that *Sarm1* deletion does not suppress gross brain atrophy secondary to TDP-43^Q331K^. However, measuring total brain weights may not be a sensitive enough method to find subtle differences in brain weight, and cannot be used to identify regional volume loss. We therefore conducted an ex vivo*,* in loco brain magnetic resonance imaging (MRI) study in a separate group of female mice. This analysis confirmed that both the Q331K-*Sarm1*^*+/−*^ (505 ± 4 mm^3^) and Q331K-*Sarm1*^*−/−*^ mice (508 ± 3 mm^3^), had significantly smaller brains than the NTG mice (534 ± 5 mm^3^), and also found no significant difference in total brain volumes between Q331K-*Sarm1*^*+/−*^ and Q331K-*Sarm1*^*−/−*^ mice (Fig. 3c).

Further analyses of the MRI data were performed to identify region-specific effects of TDP-43^Q331K^ and *Sarm1* deletion by measuring regional brain volumes and diffusivity parameters (Fractional Anisotropy (FA) and mean diffusivity (MD)) (Fig. 3d, e, Additional file 2a,b, Additional file 5: Table S1). We observed prominent decreases in regional brain volumes primarily located in temporal-equivalent and hippocampal regions of the brain, which were accompanied by much smaller changes in FA and no significant effects on MD (Fig. 3d, e, Additional file 5: Table S1). A Region of Interest (ROI) analysis compared in more detail the changes in volume and FA in specific brain regions as outlined in the DSURQE mouse atlas (Additional file 5: Table S1). Significantly reduced volume was observed in several areas including the insular region (6.77%), dorsolateral entorhinal cortex (11.65%), perirhinal cortex (14.04%), amygdala (9.7%), hippocampal CA3 (6.69%), and pre-para subiculum (7.77%) in Q331K-*Sarm1*^*+/−*^ mice compared to NTG mice. This indicates that the greatest brain volume loss occurred preferentially in temporal lobe-equivalent regions of TDP-43^Q331K^ mice (Additional file 2a). Decreased FA was observed in several white matter tracts including the anterior commissure (4.1%), stria terminalis (3.2%) internal capsule (1.1%) and facial nerve (3.5%), while several grey matter temporal regions showed an increase in FA including the ectorhinal cortex (2.11%), perirhinal cortex (1.68%), and insular region (3.00%) in Q331K-*Sarm1*^*+/−*^ mice (Additional file 5: Table S1). Overall, however, there were no significant regional differences in brain volume or FA between Q331K-*Sarm1*^*+/−*^ and Q331K-*Sarm1*^*−/−*^ mice (Additional file 2b).

To investigate the cellular cause of the changes observed with MRI in TDP-43^Q331K^ mice, YFP-expressing neurons in the cortex were counted. Neuronal numbers were significantly decreased (*p* = 0.0009) in the entorhinal cortex of Q331K-*Sarm1*^*+/−*^ mice, which contrasts with no significant loss of neurons in the motor cortex (Fig. 3f, g). Neuron numbers were not significantly different between Q331K-*Sarm1*^*+/−*^ and Q331K-*Sarm1*^*−/−*^ mice in either brain region. Collectively, these studies demonstrate that *Sarm1* deletion does not influence regional brain atrophy or neuronal loss caused by TDP-43^Q331K^, and that temporal-lobe equivalent brain regions are more significantly affected by TDP-43^Q331K^ overexpression than the primary motor cortex.

### TDP-43^Q331K^-mediated dendritic spine defects in the motor cortex are suppressed by Sarm1 deletion

**Fig. 4:**
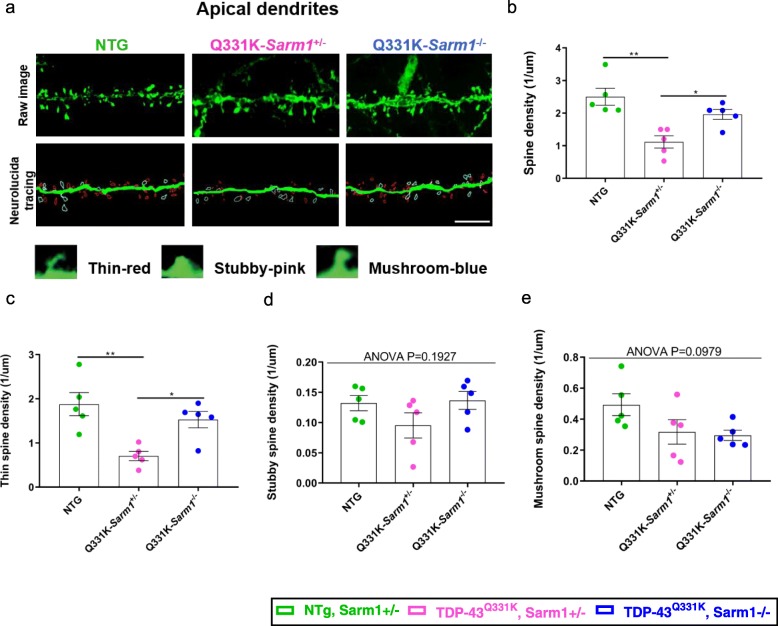
TDP-43^Q331K^-mediated dendritic spine defects in the motor cortex are partially suppressed by *Sarm1* deletion. **a**. Representative images of the Thy1-YFP motor cortex apical dendrites from Layer V cortical neurons (upper), and the corresponding Neurolucida tracing (middle; Red = thin spine, Pink = stubby spine, Blue = mushroom spine) from mice of each genotype at 10 months of age. Scale bar, 5 μm. Examples of different spine morphologies (lower) [28]. **b**. Density of apical dendritic spines per micrometre in motor cortex. ANOVA *P* = 0.0016. Pairwise comparisons: NTG vs. Q331K-*Sarm1*^+/−^: ** *P* = 0.0014; Q331K-*Sarm1*^+/−^ vs. Q331K-*Sarm1*^−/−^: * *P* = 0.0268. (**c**-**e**) Spines were classified either as mushroom, stubby or thin type according their morphologic features. Density of each type of apical dendritic spines per millimetre in motor cortex. **c**. Thin spine density: ANOVA *P* = 0.0034. Pairwise comparisons: NTG vs. Q331K-*Sarm1*^+/−^: ** *P* = 0.0034; Q331K-*Sarm1*^+/−^ vs. Q331K-*Sarm1*^−/−^: * *P* = 0.0227. **d**. Stubby spine density E. Mushroom spine density. For (**b**-**e**) (*n* = 5 mice per genotype); one-way ANOVA followed by Holm-Sidak *post-hoc* test for pairwise comparisons; error bars represent mean ± s.e.m

Synaptic degeneration and dendritic spine loss are an early feature of neurodegenerative conditions including dementias [32]. TDP-43 plays a crucial role in the formation and turnover of dendritic spines, as manipulation of TDP-43 expression causes significant changes in spine morphology and density in cortical neurons [21, 29, 33]. SARM1 also regulates synaptic plasticity and dendritic spine growth [11, 43]. Although our studies of the brain demonstrated that TDP-43^Q331K^-induced cortical atrophy and neuronal loss were not suppressed by *Sarm1* deletion, we hypothesised that *Sarm1* deletion could still mitigate dendritic spine loss in mutant mice. We therefore examined spine density and shape in layer V of the motor cortex in TDP-43^Q331K^ and NTG mice, focussing specifically on distal branches of apical dendrites, as they form abundant arbours rich in spines (Fig. 4a). TDP-43^Q331K^ expression reduced spine density to only 45% that of NTG mice (*p* = 0.0014) but this was significantly improved in Q331K-*Sarm1*^*−/−*^ mice to 78% of NTG (*p* = 0.0268) (Fig. 4b). Analyses of individual spine volume and surface area demonstrated no significant differences between any genotypes (Additional file 3a,b). Morphologically, dendritic spines can be classified into three main subsets: thin, stubby, and mushroom [34, 68]. While thin spines are usually transient with a rapid turnover, mature stubby and mushroom spines establish stable connections [9, 35]. To determine if the alterations in dendritic spine densities were associated with specific morphological subsets, we calculated the density of thin, stubby, or mushroom shaped spines (Fig. 4c-e). The density of thin spines in Q331K-*Sarm1*^*+/−*^ mice was reduced to 38% that of NTG mice (*p* = 0.0034), while stubby and mature spines were not significantly altered. However, Q331K-*Sarm1*^*−/−*^ mice demonstrated a higher density of thin spines, reaching 81% of the density seen in NTG mice (*p* = 0.0227). Collectively, these results indicate that *Sarm1* deletion significantly suppresses dendritic spine loss caused by TDP-43^Q331K^, preferentially maintaining a population of thin spines.

### Sarm1 deletion attenuates the pre-weaning loss of male TDP-43^Q331K^ mice but does not influence age-related behavioural impairments

**Fig. 5:**
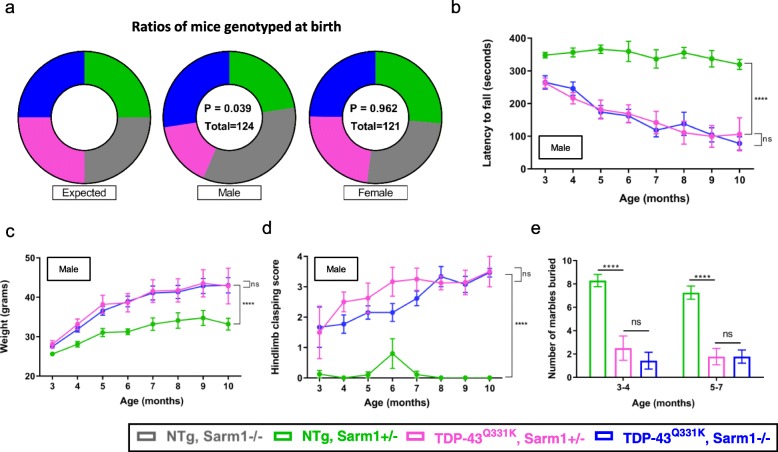
*Sarm1* deletion attenuates the pre-weaning loss of male TDP-43^Q331K^ mice but does not influence age-related behavioural impairments. **a**. Ratios of mice genotyped at birth (all of which were successfully weaned) broken down by gender. Female (χ^2^ = 0.289, d.f. = 3, *P* = 0.962), Male (χ^2^ = 8.387, d.f. = 3, *P* = 0.039); Chi square test. **b**. Latency to fall of male transgenic mice on accelerating rotarod (*n* = 5–10 NTG; *n* = 4–8 Q331K-*Sarm1*^+/−^; *n* = 6–13 Q331K-*Sarm1*^−/−^ mice per genotype). Fixed effects (Age x Genotype) *P* < 0.0001. Pairwise comparisons: Q331K-*Sarm1*^+/−^ vs. Q331K-*Sarm1*^−/−^: ns *P* = 0.6873. **c**. Weights of male mice (*n* = 4–10 NTG; *n* = 4–8 Q331K-*Sarm1*^+/−^; *n* = 13 Q331K-*Sarm1*^−/−^ mice per genotype). Fixed effects (Age x Genotype) *P* = 0.0113. Pairwise comparisons: Q331K-*Sarm1*^+/−^ vs. Q331K-*Sarm1*^−/−^: ns *P* = 0.8984. **d**. Score of male mice on hindlimb clasping test (*n* = 5–10 NTG; *n* = 4–8 Q331K-*Sarm1*^+/−^; *n* = 3–13 Q331K-*Sarm1*^−/−^ mice per genotype). Fixed effects (Age x Genotype) *P* = 0.0003. Pairwise comparisons: Q331K-*Sarm1*^+/−^ vs. Q331K-*Sarm1*^−/−^: ns *P* = 0.0764. For (**b**-**d**) Mixed-effects analysis followed by Holm-Sidak *post-hoc* test for pairwise comparisons. **e**. Number of marbles buried at different ages (*n* = 7–12 NTG; *n* = 4–9 Q331K-*Sarm1*^+/−^; *n* = 7–18 Q331K-*Sarm1*^−/−^ mice per genotype). ANOVA Genotype *P* < 0.0001. Pairwise comparisons: 3–4 months, Q331K-*Sarm1*^+/−^ vs. Q331K-*Sarm1*^−/−^: ns *P* = 0.4154; 5–7 months, Q331K-*Sarm1*^+/−^ vs. Q331K-*Sarm1*^−/−^: ns *P* > 0.9999. Two-way ANOVA followed by Holm-Sidak *post-hoc* test for pairwise comparisons. For (**b**-**e**) ****P < 0.0001; error bars represent mean ± s.e.m

Having determined that *Sarm1* deletion ameliorated TDP-43^Q331K^-induced motor neuron degeneration and dendritic spine loss we bred larger cohorts of mice for behavioural studies. During breeding we found that female mice surviving to at least 10 days of age were present at Mendelian ratios (Fig. 5a). However, fewer males survived to this age carrying the TDP-43^Q331K^ transgene than would be expected by Mendelian inheritance laws (44%, 54/124). In contrast, a higher-than-expected proportion of male mice survived that were *Sarm1*^−/−^ (61%, 76/124), and more specifically, a significant majority (63%, 34/54) of males with a TDP-43 transgene were *Sarm1*^−/−^. This suggests that successful embryonic and/or early post-natal development of male offspring is inhibited by TDP-43^Q331K^ overexpression, and that this effect is mitigated by *Sarm1* deletion.

To determine if Sarm1 signalling contributes to behavioural deficits caused by TDP-43^Q331K^ motor function was assayed using Rotarod. From 3 months of age all male TDP-43^Q331K^ mice demonstrated a progressive decline in motor performance compared to their NTG littermates, however, no significant difference was seen between Q331K-*Sarm1*^*−/−*^ and Q331K-*Sarm1*^*+/−*^ mice (Fig. 5b). Interestingly, TDP-43^Q331K^ mice gained significantly more weight than NTG littermates from as early as 3 months of age. Again, no significant difference was seen between Q331K-*Sarm1*^*−/−*^ and Q331K-*Sarm1*^*+/−*^ male mice (Fig. 5c). Similar observations were made in female mice (Additional file 4a, b). Given that weight gain is likely to impair Rotarod performance, weight-independent motor deficits in males were measured by hindlimb clasping, which is thought to be a measure of spastic motor impairment [36, 86]. From 3 months of age, greater clasping was observed in TDP-43^Q331K^ compared to NTG mice with no difference seen between Q331K-*Sarm1*^*−/−*^ and Q331K-*Sarm1*^*+/−*^ mice (Fig. 5d). To examine cognitive function we used the marble-burying assay, a measure of innate digging behaviour [74, 82]. Up to 7 months of age, NTG mice buried over 75% of marbles. However, Q331K-*Sarm1*^*+/−*^ mice demonstrated significantly attenuated digging behaviour with only ~25% of marbles being buried from as early as 3 months of age, and declining further by  7 months of age, indicating striking cognitive impairment (Fig. 5e). This deficit was not reversed by *Sarm1* deletion. Collectively, these results indicate that TDP-43^Q331K^ causes early and prominent changes in weight, motor and cognitive performance, which are not attenuated by *Sarm1* deletion.

## Discussion

Here, we have shown that by deleting *Sarm1* from a TDP-43^Q331K^ transgenic mouse model of ALS-FTD it is possible to significantly attenuate motor axon, NMJ and motor neuron cell body degeneration. *Sarm1* deletion appeared to protect motor neuron cell bodies to a greater extent than motor axons, which were in turn protected more so than NMJs (compare Fig. 1c, e and 2c). An underlying mechanism for this could be that motor neurons are reliant on neurotrophic support from distal targets of innervation for continued survival [59, 64, 76]. By preserving the physical link between the cell body and the target muscle, *Sarm1* deletion may improve cell body survival by helping to maintain retrograde trophic support. Collectively, these findings are in keeping with the hypothesis that ALS is a dying-back disease in which the most distal compartments of motor neurons (the NMJs and axons) are the most vulnerable in disease.

Importantly, our study design used *Sarm1* hemizygosity to enable comparison with littermate controls without the need for an excessively large breeding program. The inability of *Sarm1* hemizygosity to preserve severed sciatic nerves for up to 2 weeks supports this approach. However, it cannot be ruled out that *Sarm1* hemizygosity is partially protective in some circumstances. This raises the possibility that in the present study we may be underestimating the protective capacity of *Sarm1* deletion as we did not utilise mice that were *Sarm1*^*+/+*^ as controls.

This study used a TDP-43^Q331K^ transgenic mouse previously described as a model of ALS [4]. Interestingly, we observed several characteristics that were not previously reported for this model and which are reminiscent of FTD. We noted that TDP-43^Q331K^ mice gained significant weight compared to NTG mice. Mutants were also strikingly cognitively impaired and demonstrated significant brain atrophy from an early stage. No weight gain or cognitive dysfunction was previously described in this mouse model [4]. These differences could be because mice bred for this study were on a different background to that previously described. Although food intake was not measured, the excessive weight gain that mutants displayed could be due to hyperphagia, which is a feature of human FTD, and which we previously described in TDP-43^Q331K^ knock-in mice [82]. It also remains possible that this weight gain is due to direct effects of TDP-43 overexpression on lipid metabolism [12, 70].

In keeping with findings in humans, MRI demonstrated prominent brain atrophy in areas corresponding to the temporal lobe in these mice, and we histologically corroborated a greater neuronal loss in the entorhinal cortex than in the motor cortex. This is interesting, because studies in humans have shown that the temporal lobe can be significantly affected in ALS patients even without clinical evidence of dementia or temporal lobe-specific dysfunction (Loewe et al. Sci Rep 2017). While this study found no evidence of changes in MD, decreased FA was detected in several white matter tracts further matching the tractography findings in human patients [56]. Small but significant increases in FA in the temporal grey matter regions that also feature volume loss are difficult to interpret, but may be linked to a combination of alterations in glial and fibre density [71]. Collectively, these observations suggest that this TDP-43^Q331K^ transgenic mouse recapitulates features of FTD as well as ALS.

Of particular relevance to FTD, mutant TDP-43 has previously been shown to cause cortical dendritic spine abnormalities that are associated with attenuated neuronal transmission [21, 29]. In keeping with this, our study revealed significant brain atrophy and dendritic spine loss in TDP-43^Q331K^ mice. Furthermore, our study is the first to demonstrate that this dendritic spine degeneration can be mitigated by deletion of *Sarm1*. This is notable as Wallerian and Wallerian-like degeneration has only previously been linked with axonal, NMJ and post-synaptic integrity. Our results now suggest that Wallerian pathways are relevant to post-synaptic compartments of neurons, although it is also possible that the increase in dendritic spines following *Sarm1* deletion is secondary to the preservation of presynaptic nerve terminals that synapse onto the spines.

Interestingly, TDP-43^Q331K^ overexpression phenotypes show evidence of sexual divergence. This comes from the observation that during breeding, mutant mice were underrepresented amongst males but not females. This is in keeping with our previous observations in TDP-43^Q331K^ knock-in mice, in which mutant males but not females were present at a lower frequency than would be expected by Mendelian laws of inheritance [82]. These phenomena are also more generally consistent with the higher incidence of ALS in male patients [48, 75]. Furthermore, we also found that amongst males there were significantly more Q331K-*Sarm1*^*−/−*^ mice than Q331K-*Sarm1*^*+/−*^ mice (Fig. 1b). This suggests that TDP-43^Q331K^ influences nervous system development in a way that is attenuated by *Sarm1* deletion. We speculate that this beneficial effect of *Sarm1* deletion may occur in utero by influencing Wallerian-like degeneration in a manner similar to that observed during the rescue of CNS nerve tracts and peripheral nerve axons in embryos lacking *Nmnat2* [26].

Despite the suppression of neurodegeneration by *Sarm1* deletion, this was insufficient to cause behavioural improvements in TDP-43^Q331K^ overexpressing mice. This could be because this mouse model demonstrated very early and marked brain and muscle atrophy, which may be difficult to reverse. A similar explanation may underlie the apparent lack of efficacy of *Sarm1* deletion in a mutant SOD1 mouse model of ALS, which demonstrates rapid disease onset and progression [57], and in our previous study in *Drosophila* in which even clonal TDP-43^Q331K^ overexpression causes early and aggressive neurodegeneration [69]. We speculate that under less extreme degrees of cellular disintegration, and in more physiological models, which are likely to be more reflective of early disease states in patients with ALS-FTD, *Sarm1* deletion may have a greater ability to attenuate behavioural and motor dysfunction. Further support of this comes from human genome-wide studies that have identified an association between genetic variants at the human *SARM1* locus and the risk of developing ALS [22]. It remains to be determined if these genetic variants influence SARM1 expression, but if so then this would further support a mechanistic link between TDP-43-mediated toxicity in sporadic ALS and Wallerian-like degeneration.

## Conclusions

In conclusion, our results indicate that a Sarm1 dependent pathway contributes to TDP-43^Q331K^-mediated motor neuron, motor axon, NMJ and cortical spine degeneration in vivo. Anti-SARM1 therapies therefore have potential as a treatment for diseases of the ALS-FTD spectrum.

## Additional files

Additional file 1: Gastrocnemius muscle weights in male and female mice. (PDF 720 kb)

Additional file 2: Additional MRI data. (PDF 4210 kb)

Additional file 3: Dendritic spine morphology in motor cortex. (PDF 459 kb)

Additional file 4: Behavioural characterisation of female mice. (PDF 747 kb)

Additional file 5: **Table S1.** Results of ROI analyses of volume and FA changes in different brain 121 regions comparing the mean of each genotype with that of every other group. (PDF 602 kb)

## Data Availability

The authors will make all data available to readers upon reasonable request.

## Abbreviations

ALS: Amyotrophic lateral sclerosis
FTD: Frontotemporal dementia
NAD: Nicotinamide adenine dinucleotide
*Nmnat*: Nicotinamide mononucleotide adenylyltransferase
*Sarm1*: Sterile alpha and TIR motif-containing protein 1
SNP: Single nucleotide polymorphism
TDP-43: TAR DNA-binding protein 43
*Wld*^*S*^: Wallerian degeneration slow
