# Supplementary material for: Sarm1 deletion suppresses TDP-43-linked motor neuron degeneration and cortical spine loss
Source: Acta Neuropathol Commun. 2019 Oct 28;7:166. doi: 10.1186/s40478-019-0800-9 (PMC6819591; doi:10.1186/s40478-019-0800-9)
Supplement: Supplementary file 1 — Gastrocnemius muscle weights in male and female mice. (PDF 720 kb) [file 40478_2019_800_MOESM1_ESM.pdf]

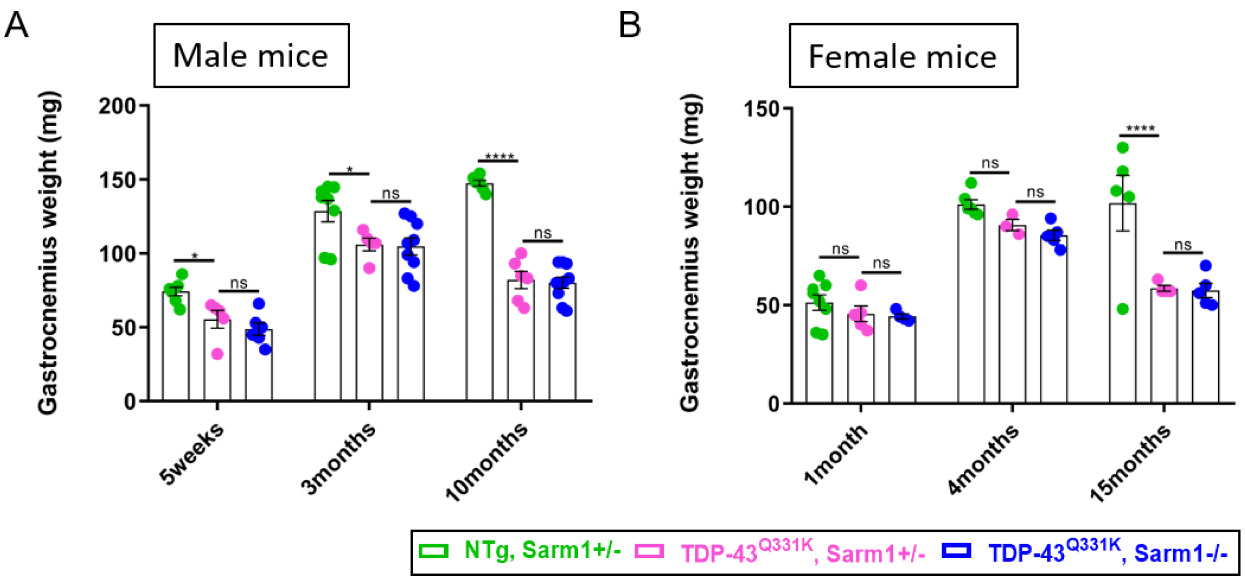


**Additional file 1 Gastrocnemius muscle weights in male and female mice**

**A.** Gastrocnemius muscle weights of male mice at different time points (n=6-8 NTG; n=5-6 Q331K-*Sarm1*$^{+/-}$; n=6-10 Q331K-*Sarm1*$^{-/-}$). ANOVA Interaction P<0.0001. Pairwise comparisons: 5 weeks, NTG vs. Q331K-*Sarm1*$^{+/-}$: * P=0.0427; Q331K-*Sarm1*$^{+/-}$ vs. Q331K-*Sarm1*$^{-/-}$: ns P=0.4180; 3 months, NTG vs. Q331K-*Sarm1*$^{+/-}$: * P=0.0106; Q331K-*Sarm1*$^{+/-}$ vs. Q331K-*Sarm1*$^{-/-}$: ns P=0.8614; 10 months, Q331K-*Sarm1*$^{+/-}$ vs. Q331K-*Sarm1*$^{-/-}$: ns P=0.7990. **B.** Gastrocnemius muscle weights of female mice at different time points (n=5-8 NTG; n=3-5 Q331K-*Sarm1*$^{+/-}$; n=4-5 Q331K-*Sarm1*$^{-/-}$). ANOVA Interaction P=0.0063. Pairwise comparisons: 1 month, NTG vs. Q331K-*Sarm1*$^{+/-}$: ns P=0.7560; Q331K-*Sarm1*$^{+/-}$ vs. Q331K-*Sarm1*$^{-/-}$: ns P=0.8753; 4 months, NTG vs. Q331K-*Sarm1*$^{+/-}$: ns P=0.4391; Q331K-*Sarm1*$^{+/-}$ vs. Q331K-*Sarm1*$^{-/-}$: ns P=0.5745; 15 months, Q331K-*Sarm1*$^{+/-}$ vs. Q331K-*Sarm1*$^{-/-}$: ns P=0.8982. For (A-B) two-way ANOVA followed by Holm-Sidak *post-hoc* test for pairwise comparisons; ****P<0.0001; error bars represent mean ± s.e.m.
