# Supplementary material for: Sarm1 deletion suppresses TDP-43-linked motor neuron degeneration and cortical spine loss
Source: Acta Neuropathol Commun. 2019 Oct 28;7:166. doi: 10.1186/s40478-019-0800-9 (PMC6819591; doi:10.1186/s40478-019-0800-9)
Supplement: Supplementary file 2 — Additional MRI data. (PDF 4210 kb) [file 40478_2019_800_MOESM2_ESM.pdf]

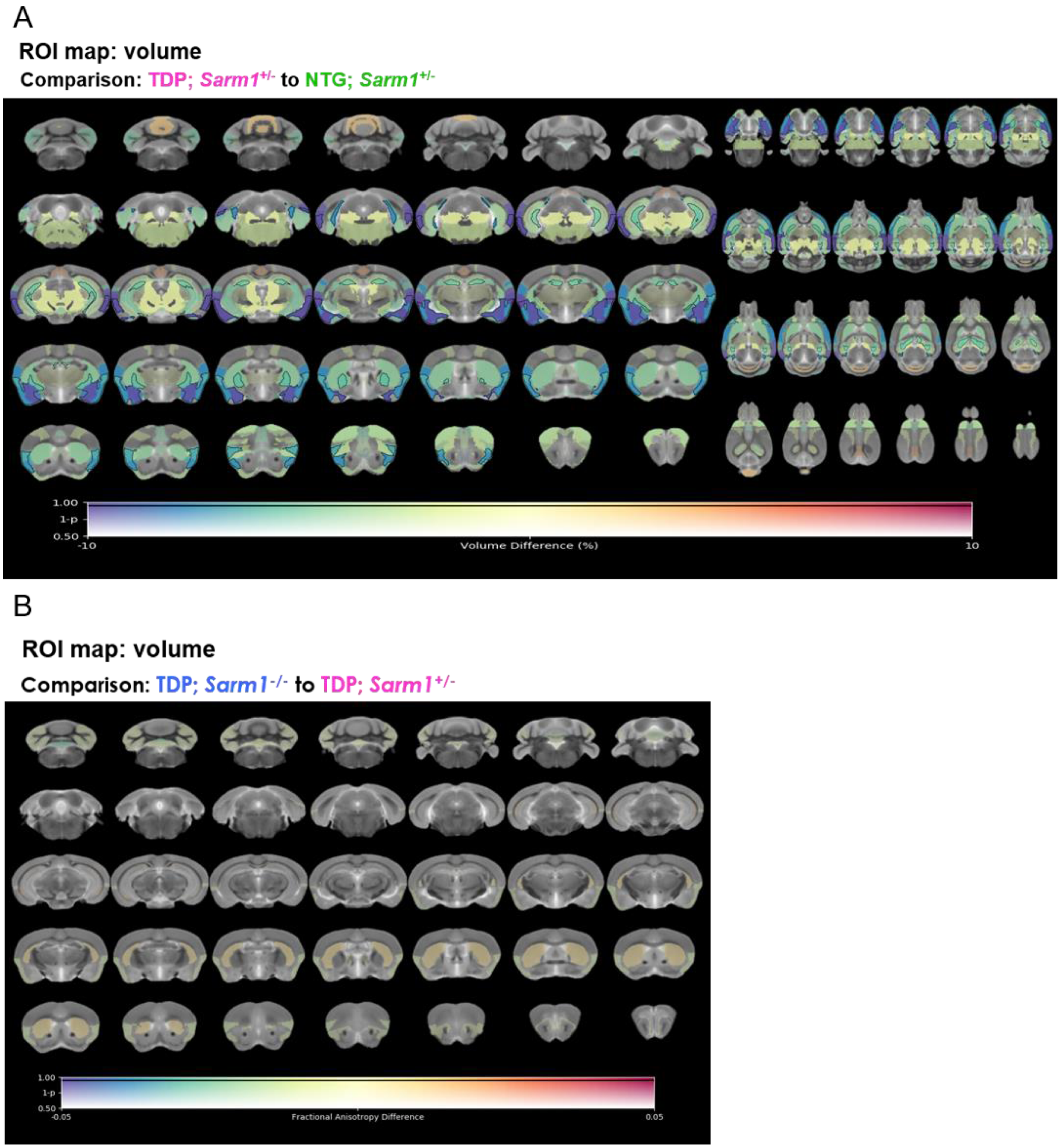

Additional file 2 Additional MRI data

A-B. MRI study-specific template (coronal and transverse) with an overlay representing ROI volume differences at 10 months of age (A) between Q331K-*Sarm1*$^{+/-}$ and NTG mice (B) between Q331K-*Sarm*$^{-/-}$ and Q331K-*Sarm1*$^{+/-}$ mice. The colour of the overlay indicates the inter-group volume difference, while the transparency indicates the statistical significance. ROIs in which FWE-corrected p value <0.05 are contoured in black
