# Supplementary material for: Sarm1 deletion suppresses TDP-43-linked motor neuron degeneration and cortical spine loss
Source: Acta Neuropathol Commun. 2019 Oct 28;7:166. doi: 10.1186/s40478-019-0800-9 (PMC6819591; doi:10.1186/s40478-019-0800-9)
Supplement: Supplementary file 3 — Dendritic spine morphology in motor cortex. (PDF 459 kb) [file 40478_2019_800_MOESM3_ESM.pdf]

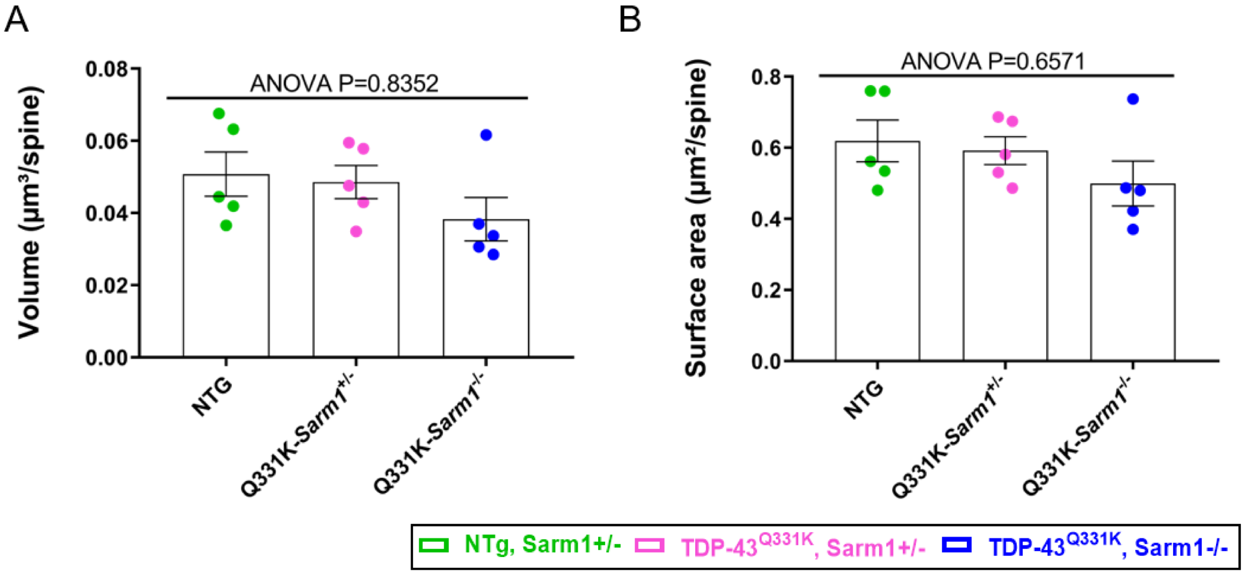

Additional file 3 Dendritic spine morphology in motor cortex

A. Volume and **B.** Surface area of individual apical dendritic spines in motor cortex from mice at 10 months of age (n=5 mice per genotype). Spine volume ANOVA P=0.8352; spine surface area ANOVA P=6571; one-way ANOVA; error bars represent mean ± s.e.m.
