# Supplementary material for: Sarm1 deletion suppresses TDP-43-linked motor neuron degeneration and cortical spine loss
Source: Acta Neuropathol Commun. 2019 Oct 28;7:166. doi: 10.1186/s40478-019-0800-9 (PMC6819591; doi:10.1186/s40478-019-0800-9)
Supplement: Supplementary file 4 — Behavioural characterisation of female mice. (PDF 747 kb) [file 40478_2019_800_MOESM4_ESM.pdf]

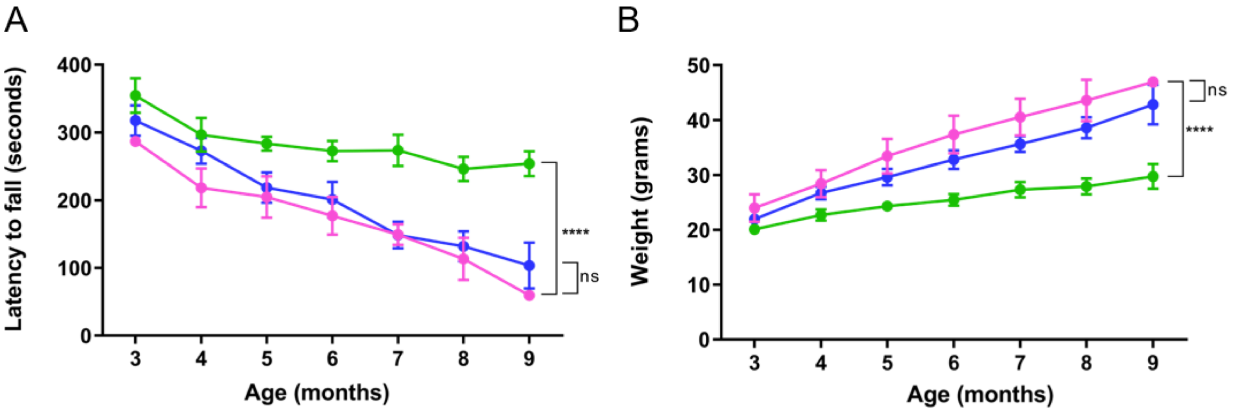

Additional file 4 Behavioural characterisation of female mice

A. Latency to fall of female transgenic mice on accelerating rotarod (n=4-10 NTG; n=3-12 Q331K-*Sarm1*$^{+/-}$; n=5-12 Q331K-*Sarm1*$^{-/-}$ mice per genotype). Fixed effects (Age x Genotype) P=0.0232. Pairwise comparisons: Q331K-*Sarm1*$^{+/-}$ vs. Q331K-*Sarm1*$^{-/-}$: ns P=0.2352. **B.** Weights of female mice (n=4-10 NTG; n=3-5 Q331K-*Sarm1*$^{+/-}$; n=5-12 Q331K-*Sarm1*$^{-/-}$ mice per genotype). Fixed effects (Age x Genotype) P<0.0001. Pairwise comparisons: Q331K-*Sarm1*$^{+/-}$ vs. Q331K-*Sarm1*$^{-/-}$: ns P=0.1248; two-way ANOVA followed by Holm-Sidak *post-hoc* test for pairwise comparisons. For (A-B) ****P<0.0001; error bars represent mean ± s.e.m.
