# Supplementary material for: Sarm1 deletion suppresses TDP-43-linked motor neuron degeneration and cortical spine loss
Source: Acta Neuropathol Commun. 2019 Oct 28;7:166. doi: 10.1186/s40478-019-0800-9 (PMC6819591; doi:10.1186/s40478-019-0800-9)
Supplement: Supplementary file 5 — Table S1. Results of ROI analyses of volume and FA changes in different brain 121 regions comparing the mean of each genotype with that of every other group. (PDF 602 kb) [file 40478_2019_800_MOESM5_ESM.pdf]

Additional table 1 Results of ROI analyses of volume and FA changes in different brain regions comparing the mean of each genotype with that of every other group

Results of ROI analyses (Volume):

BRAIN REGIONS (VOLUME)	Volume diff (%)	FDR adjusted p value	Volume diff (%)	FDR adjusted p value
	Q331K-*Sarm1*$^{+/-}$ to NTG		*Q331K-Sarm1*$^{-/-}$ to Q331K-*Sarm1*$^{+/-}$	
DORSOLATERAL ENTORHINAL CORTEX	-11.6538	0.0223	-3.3313	0.9981
VENTRAL NUCLEUS OF THE ENDOPIRIFORM CLAUSTRUM	-7.9053	0.0300	-2.1476	0.9981
DORSAL INTERMEDIATE ENTORHINAL CORTEX	-10.1338	0.0332	-3.1552	0.9981
PODG	-5.9565	0.0387	-0.0686	0.9981
GRDG	-5.7484	0.0332	0.3160	1.0000
ROSTRAL AMYGDALOPIRIFORM AREA	-11.8198	0.0199	0.1169	0.9981
MODG	-4.9296	0.0332	0.0173	0.9981
PERIRHINAL CORTEX	-14.0439	0.0395	-2.0329	0.9981
AMYGDALA	-9.7262	0.0223	-1.3559	0.9981
GLOBUS PALLIDUS	-5.8643	0.0395	2.9526	0.9981
POSTEROLATERAL CORTICAL AMYGDALOID AREA	-8.7475	0.0199	0.9273	0.9981
CA3PY INNER	-6.6886	0.0332	1.7009	0.9981
PIRIFORM CORTEX	-7.6397	0.0332	0.3921	1.0000
CLAUSTRUM: DORSAL PART	-9.6765	0.0332	2.0760	0.9981

**INSULAR REGION: NOT SUBDIVIDED**	-6.7702	0.0332	-0.3478	1.0000
**AMYGDALOPIRIFORM TRANSITION AREA**	-13.6769	0.0395	4.9936	0.9981
**PRE-PARA SUBICULUM**	-7.7679	0.0465	-0.6426	0.9981
**MAMMILOTHALAMIC TRACT**	-1.5175	0.2651	-3.4164	0.9981
**STRIATUM**	-4.6261	0.1235	-0.6352	1.0000
**CAUDOMEDIAL ENTORHINAL CORTEX**	-4.4692	0.1561	-2.3356	0.9981
**ECTORHINAL CORTEX**	-10.8688	0.0642	2.5069	0.9981
**DORSAL NUCLEUS OF THE ENDOPIRIFORM**	-7.1351	0.1101	0.3102	0.9981
**SUBICULUM**	-3.7538	0.1561	0.2727	0.9981

Results of ROI analyses (FA):

**BRAIN REGIONS (FA)**	**FA diff (%)**	**FDR adjusted p value**	**FA diff (%)**	**FDR adjusted p value**
	**Q331K-Sarm1$^{+/-}$ to NTG**		***Q331K-Sarm1*$^{-/-}$ to Q331K-*Sarm1*$^{+/-}$**	
**ANTERIOR COMMISSURE: PARS POSTERIOR**	-4.0999	0.0447	0.6890	0.6975
**CEREBRAL PEDUNCLE**	-2.4513	0.0447	-0.0298	0.8237
**TEMPORAL ASSOCIATION AREA**	2.0450	0.0449	-0.7689	0.7262
**ECTORHINAL CORTEX**	2.1129	0.0449	-0.9783	0.7575
**STRIA TERMINALIS**	-3.2239	0.0457	0.4587	0.9239

**PRIMARY AUDITORY CORTEX**	2.0389	0.0449	0.0425	0.9805
**DORSOLATERAL ENTORHINAL CORTEX**	2.7990	0.0457	-0.4842	0.8237
**FACIAL NERVE (CRANIAL NERVE 7)**	-3.5496	0.0457	-0.8996	0.8039
**MEDIAL ENTORHINAL CORTEX**	2.2699	0.0449	-0.0160	0.8039
**INTERNAL CAPSULE**	-1.1202	0.0449	-0.1370	0.8237
**INSULAR REGION: NOT SUBDIVIDED**	3.0023	0.0457	-1.4650	0.3051
**MEDIAL SEPTUM**	-0.9700	0.0486	0.0516	0.8237
**INSULAR REGION: NOT SUBDIVIDED**	3.0023	0.0457	-1.4650	0.3051
**MEDIAL SEPTUM**	-0.9700	0.0486	0.0516	0.8237
**SECONDARY AUDITORY CORTEX: VENTRAL AREA**	1.2868	0.0615	-0.1850	0.9805
**FASCICULUS RETROFLEXUS**	-2.4414	0.0615	0.8117	0.9081
**SLU**	0.9989	0.1588	0.7993	0.6975
**PRIMARY SOMATOSENSORY CORTEX: UPPER LIP REGION**	0.8457	0.1588	0.9299	0.6975
**SUBEPENDYMALE ZONE / RHINOCELE**	-2.1028	0.1932	-0.8214	0.6572
**CA3RAD**	1.1479	0.3220	0.5122	0.6572
**POSTERIOR COMMISSURE**	1.2645	0.3429	0.8172	0.6572
**STRIA MEDULLARIS**	-0.3826	0.4300	-0.9390	0.3703
**LOBULE 1-2 WHITE MATTER**	-1.0779	0.6963	-1.8440	0.3703
